# Interferon regulatory factor-7 modulates experimental autoimmune encephalomyelitis in mice

**DOI:** 10.1186/1742-2094-8-181

**Published:** 2011-12-23

**Authors:** Mohammad Salem, Jyothi T Mony, Morten Løbner, Reza Khorooshi, Trevor Owens

**Affiliations:** 1 Department of Neurobiology Research, Institute of Molecular Medicine, University of Southern Denmark, Odense, Denmark

**Keywords:** IRF7, type I IFN, EAE, inflammation, central nervous system, chemokines, cytokines

## Abstract

**Background:**

Multiple sclerosis (MS) is an inflammatory disease of the central nervous system (CNS) with unknown etiology. Interferon-β (IFN-β), a member of the type I IFN family, is used as a therapeutic for MS and the IFN signaling pathway is implicated in MS susceptibility. Interferon regulatory factor 7 (IRF7) is critical for the induction and positive feedback regulation of type I IFN. To establish whether and how endogenous type I IFN signaling contributes to disease modulation and to better understand the underlying mechanism, we examined the role of IRF7 in the development of MS-like disease in mice.

**Methods:**

The role of IRF7 in development of EAE was studied by immunizing IRF7-KO and C57BL/6 (WT) mice with myelin oligodendrocyte glycoprotein using a standard protocol for the induction of EAE. We measured leukocyte infiltration and localization in the CNS using flow cytometric analysis and immunohistochemical procedures. We determined levels of CD3 and selected chemokine and cytokine gene expression by quantitative real-time PCR.

**Results:**

IRF7 gene expression increased in the CNS as disease progressed. IRF7 message was localized to microglia and infiltrating leukocytes. Furthermore, IRF7-deficient mice developed more severe disease. Flow cytometric analysis showed that the extent of leukocyte infiltration into the CNS was higher in IRF7-deficient mice with significantly higher number of infiltrating macrophages and T cells, and the distribution of infiltrates within the spinal cord was altered. Analysis of cytokine and chemokine gene expression by quantitative real-time PCR showed significantly greater increases in CCL2, CXCL10, IL-1β and IL17 gene expression in IRF7-deficient mice compared with WT mice.

**Conclusion:**

Together, our findings suggest that IRF7 signaling is critical for regulation of inflammatory responses in the CNS.

## Background

Multiple sclerosis (MS) is a chronic inflammatory demyelinating disease of the central nervous system (CNS), which is likely triggered by infection or other environmental events [1,2]. Experimental autoimmune encephalomyelitis (EAE) is an animal model for MS that is induced by immunization with myelin antigens [3]. In both MS and EAE demyelinating lesions are accompanied by T cell and macrophage infiltration [2,3].

The first clinically approved therapy for MS was IFN-β [4,5], a member of the type I IFN family that also includes multiple IFN-α subtypes. Type I IFNs are classically induced by viral infection and act through a common receptor, IFNAR [6]. The transcription factor IRF7 is constitutively expressed at low levels in the cytoplasm [7,8], and becomes activated by innate receptor signaling, resulting in translocation to the nucleus and induction of type I IFN [9]. Type I IFN signaling leads to further induction of IRF7, so creating a feed-forward loop to amplify production of type I IFN. IRF7 may therefore represent a link between innate receptor and type I IFN signaling. Consequently, changes in IRF7 function may affect processes regulated by type I IFN. Mice lacking IRF7 are deficient in type I IFN responses and consequently lack innate responsiveness to viruses [10,11].

In addition to their antiviral function, type I IFNs play a critical role in the regulation of inflammation in the CNS [12]. Mice lacking either IFNβ or IFNAR develop more severe EAE, with increased CNS infiltration [13-15]. Recent evidence suggests that type I IFN may be produced within the CNS, in response to inflammation or injury, and that signaling through IFNAR modulates leukocyte infiltration [7,8,16]. We have shown that synaptic degeneration-induced IRF7 increase in the CNS is IFNAR-dependent [8].

The signaling pathways mediating production and effect of type I IFN in the CNS remain uncertain. Here we analyze the role of IRF7 in EAE, and show that mice lacking this transcription factor develop more severe EAE, with increased CNS infiltration. This implicates IRF7 as a key signaling intermediate in modulation of autoimmune demyelinating disease. Due to its regulatory action on type I IFN signaling, IRF7 therefore represents an important factor that regulates development of CNS autoimmune diseases, such as MS.

## Materials and methods

### Animals

IRF7-KO mice on C57BL/6 (B6) background were purchased from Riken BioResource Center (Tsukuba, Japan) and maintained as a breeding colony. Control wild-type B6 mice, which have been shown to be appropriate controls for EAE studies [17], were obtained from Taconic (RY, Denmark). Mice were provided with food and water *ad libitum*. All experiments were approved by the Danish Justice Ministry Committee on Animal Research (Approval Number 2009/561-1724).

### Induction of EAE and Clinical Evaluation

To induce EAE, adult female IRF7-KO and control B6 mice were subcutaneously immunized with 35-55 myelin oligodendrocyte glycoprotein (35-33 MOG) peptide in complete Freund's adjuvant containing 2 mg/ml *M*. tubercolosis in the flanks. In addition, mice received intraperitoneal injections with 200 μl pertussis toxin (1,5 μg/ml) (Sigma-Aldrich, Brøndby, Denmark) at the time of immunization and two days later. Mice were then caged in groups of 8 (4 WT mice with 4 IRF7-KO mice). The mice were weighed and monitored daily for clinical signs of EAE, which were scored as follows: 0, no symptoms; 1, Weak or hooked tail; 2, Floppy tail; 3, 2 + hind limb paresis (weak hind limbs-assessed by the animal's slowness or splaying limbs when walking or unsteady walk in the cage or on the lid of the cage), Grade 4: 3 + very weak hind limbs or one hind limb paralysed- hind limb paresis-assessed by the animal dragging one or both hind limbs (not complete loss of tonus in one or both hind limbs); 5, 4 + unilateral hind limb paralysis (both hind limbs paralysed); 6, 5 + paresis in one forelimb. Because of ethical reasons, mice were euthanized when they reached a clinical score of 5. In the first experiment the clinical score in first euthanized 4 mice was 3-4, and all other euthanized mice had clinical score 5. At the end of experiment all remaining mice were euthanized. In the second and third experiment, half of the mice were sacrificed at day 15 and the rest either when they scored 4-5 or at the end of experiment. Mice were weighed and scored in a blinded manner.

### Tissue preparation

Mice were deeply anaesthetized and perfused intracardially with ice-cold Phosphate Buffered Saline (PBS). Spinal cords were dissected out and processed as followed:

For Histology: the tissues were placed in 4% paraformaldehyde (PFA) (Sigma-Aldrich) for 60 minutes and overnight in 1% PFA at 4°C. The tissues were then placed in 20% sucrose solution overnight, freeze-embedded in cryo-embedding (Ax-lab, Vedbæk, Denmark), cut in 16-μm cryostat sections, mounted on glass slides and stored at -80°C.

For Flow Cytometry: the tissues were placed in a plate with Hanks Balanced Salt Solution (HBSS) (Invitrogen A/S, Taastrup, Denmark) for further processing.

For *Quantitative real-time reverse transcriptase- PCR assay*: the tissues were placed in eppendorf tubes containing TRIzol (Invitrogen Life Technologies, Paisley, Scotland, UK), which were then stored in -80°C until further processing.

### Histology

To investigate the extent and distribution of histopathology Hematoxylin and Eosin staining was performed. Double Immunostaining was used to detect astrocytes and T-cells. In brief, sections were washed in PBS, followed by rinsing in PBS-0.5% Triton (Triton- X-100) (Sigma-Aldrich) (PBST) and blocked in a solution containing PBST and 3% BSA (Sigma-Aldrich). Thereafter, sections were incubated with Cy-3 conjugated mouse anti GFAP antibody (C9205, Sigma-Aldrich), and Rat anti-mouse CD3 (MCA500G, Serotec) antibodies, in order to detect astrocytes and T cells respectively. After several washes in PBST, sections were incubated with donkey anti-rat Alexa Fluor-488 antibody (Invitrogen- Molecular Probes, Taastrup, Denmark), to visualize anti-CD3 antibody. Nuclei were then stained with DAPI (Invitrogen-Molecular Probes). To test the specificity of staining, control sections were treated without primary antibody or with isotype-matched primary antibodies. Control sections displayed no staining comparable with that seen without primary antibodies (not shown). Images were acquired using an Olympus BX51 microscope (Olympus, Denmark) connected to an Olympus DP71 digital camera, and combined using Adobe Photoshop CS version 8.0 to visualize double-labeled cells.

### Flow cytometry

Single cell suspensions of spinal cords and lymph nodes (LN) were prepared by dissociation using a 70 μm cell strainer (BD Biosciences, Brøndby, Denmark). Spinal cord samples were resuspended in 37% Percoll (GE Healthcare Bio-sciences AB, Uppsala, Sweden) and centrifuged to remove myelin. Blocking was performed using Mouse Fc Block (BD Biosciences). Cells were stained with biotinylated anti-mouse CD8, FITC anti-mouse CD4 or PerCP/Cy5.5 anti-mouse CD11b and phycoerythrin (PE) anti-mouse CD45 (BD Biosciences). Data was collected on a FACS Calibur (BD Biosciences), and analyzed using Flowjo software (Tree Star, Ashland, OR).

### T cell stimulation and intracellular cytokine staining

Single cell suspensions prepared as described above were plated in 96 well plates coated with anti-mouse CD3ε (145-2C11) and cultured for 9 hours to stimulate cytokine production in T cells. GolgiPlug (BD Biosciences) was added two hours after plating. After incubation, cells were washed and stained with V500-rat anti mouse CD4 (BD), PerCP/Cy5.5 anti-mouse CD8α (Biolegend) and either allophycocyanin (APC)-anti-mouse CD196 (CCR6) (Biolegend) or biotin anti-mouse CD183 (CXCR3) (Biolegend) and APC-Streptavidin (BD Biosciences). Intracellular cytokine staining was performed using a Cytofix/Cytoperm kit (BD). PE-rat anti-mouse IL17A (BD Biosciences), PE/Cy7 anti-mouse IFNγ (Biolegend) were used to detect the cytokines. Data was collected on an LSR II (BD Biosciences), and analyzed using FACS DIVA software (BD).

### Fluorescence Activated Cell Sorting

Samples were prepared as described above and stained with V500-rat anti mouse CD4 (BD Biosciences), PerCP/Cy5.5 CD11b (Biolegend), PerCP/Cy5.5 anti-mouse CD8α (Biolegend), and either APC anti-mouse CD196 (CCR6) (Biolegend) or Biotin anti-mouse CD183 (CXCR3) (Biolegend) and APC-Streptavidin (BD Biosciences). Cells were sorted on a FACSVantage/Diva cell sorter (BD Biosciences).

### Quantitative Real-Time Reverse Transcriptase- PCR assay

Total RNA was purified using TRIzol RNA isolation reagent (Invitrogen Life Technologies) according to the manufacturer's protocol for whole tissue RNA extraction. One μg of RNA from each spinal cord sample was incubated with Moloney murine leukemia virus RT (Invitrogen Life Technologies) according to the manufacturer's protocol, using random hexamer primers. Quantitative Real-Time Reverse Transcriptase- PCR assay (Quantitative RT-PCR) were performed using ABI Prism 7300 Sequence Detection Systems (Applied Biosystems, Foster City, CA). Quantitative RT-PCR was performed for IRF7, CCL2, CXCl10, TNF-α, IL-1β, IFNγ, IL17 and CD3, using primers and probes as described previously [8,18]. 18s rRNA primers and probes (Applied Biosystems) were used as an endogenous control to account for differences in the extraction and RT of total RNA [8]. Each reaction was performed in 25 μl with TaqMan 2× Universal PCR Master Mix (Applied Biosystems), undiluted cDNA, primers, TaqMan probe, and 2× filtered sterile milliQ water. For all genes, PCR conditions were 2 minute at 50°C, 10 minutes at 95°C followed by 40 cycles each consisting of 15 seconds at 95°C and 1 minute at 60°C. To determine the relative RNA levels within the samples, standard curves for the PCR were prepared using cDNA from a reference sample and making fourfold serial dilutions. Relative expression values were then calculated by dividing the expression level of the target gene by the expression level of 18s rRNA.

### Statistical analysis

Data were analyzed by nonparametric, Mann-Whitney *t*-test using GraphPad Prism software (GraphPad Software Inc., San Diego, California, USA). A p value < 0.05 was considered to be statistically significant. Data are presented as Mean ± SEM.

## Results

### Upregulation of IRF7 gene expression in EAE

**Figure 1:**
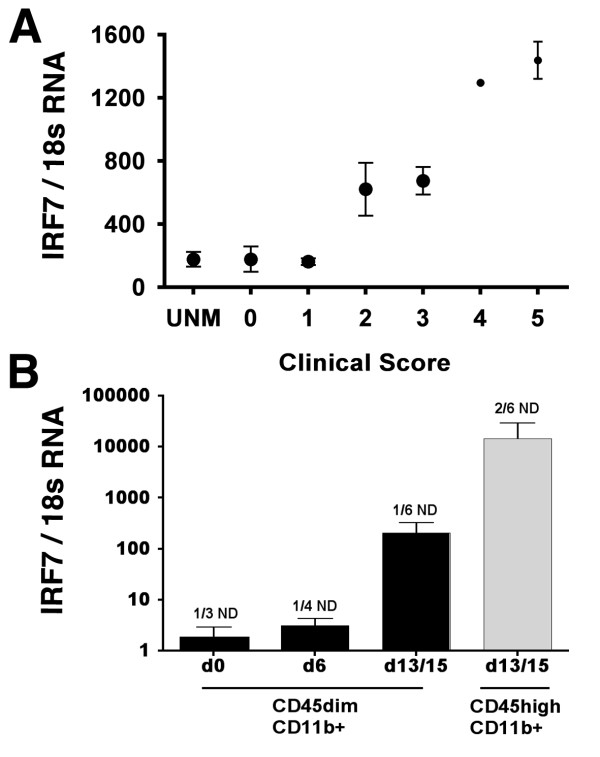
**Upregulation of IRF7 mRNA in CNS**. A) Increased IRF7 gene expression correlated with clinical score. Values on Y axis are levels of IRF7 mRNA normalized to 18S rRNA expression. Values on the × axis show clinical score. B) Increased IRF7 gene expression by CD45dimCD11b+ microglia at peak disease was not statistically significant different from levels in CNS-infiltrating CD45highCD11b+ myeloid cells. ND: not detected; UNM: unmanipulated WT B6 mice.

IRF7 gene expression was measured in spinal cords from WT mice that had been immunized with MOGp35-55+CFA. The results from three experiments are combined in Figure 1 and 1 show that induction of EAE leads to increased IRF7 gene expression. In addition the up-regulation of IRF7 mRNA correlated with the clinical score (Figure 1A). Consistent with the well-known widespread expression of Type I IFN and its response elements, as well as with previous studies [7,8,16], we found expression of IRF7 mRNA by Th1 and Th17 CD4+ T cells, and by macrophages and microglia (additional file 1 and Figure 1B). IRF7 gene expression increased in CD45dimCD11b+ microglia during the course of EAE nearly reaching the levels seen in CD45highCD11b+ myeloid cells infiltrating the CNS (Figure 1B).

### IRF7-deficient mice develop more severe EAE compared with WT

**Figure 2:**
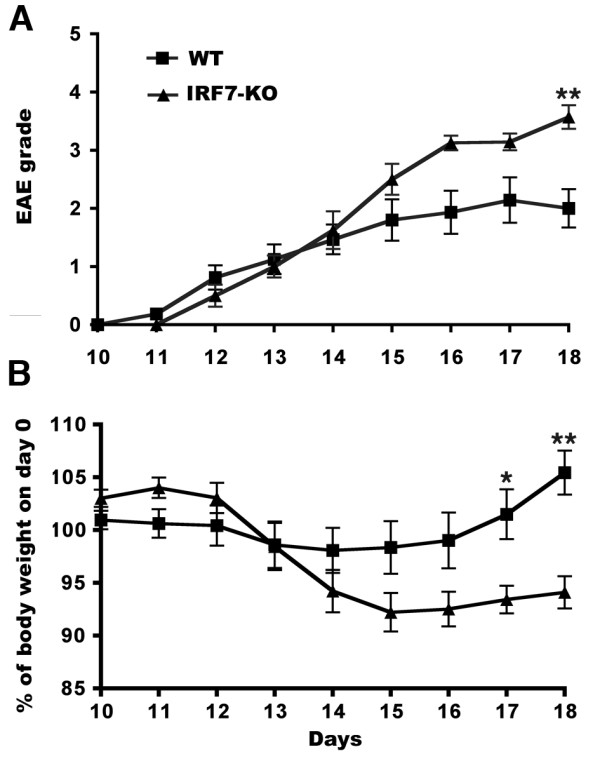
**IRF7-KO mice develop more severe EAE**. A) Clinical score of mice with EAE. IRF7 deficient mice (n = 8) developed more severe EAE than WT B6 control (n = 16) as indicated by the asterisk. B) Change in whole body weights as percent of weight one day prior to immunization (Day 0). * P < 0.05, ** p < 0.01.

To assess the role of IRF7 in EAE, we immunized IRF7-deficient mice with MOG in CFA with pertussis toxin, a standard protocol for the induction of EAE. In four independent experiments, the mean time of disease onset was not significantly different between WT (12.99 ± 1.0 day) and IRF7-KO (12.07 ± 0.7 day) (Table 1). However, the incidence of EAE differed, being 29/39 (74%) versus 28/30 (93%) in WT and IRF7-KO mice, respectively (Table 1). Nearly half of the animals with EAE were euthanized at grade 5 in the IRF7-KO group, compared to only 4/29 of those in the WT group (Table 1). Whereas the number of mice with EAE that did not achieve grade 3 was almost 50% in WT groups, less than a quarter of IRF7-KO mice failed to reach this level of severity (Table 1). Results from one experiment are shown as mean clinical scores in Figure 2A. IRF7 deficient mice developed significantly more severe EAE symptoms (Figure 2A). The need for ethical reasons to euthanize mice with severe disease disallowed study of disease progression in severely affected animals and may have obscured a statistically significant difference in severity. Increased disease severity was paralleled by significantly greater loss of whole body weight (Figure 2B).

**Table 1:** Relative incidence, onset, and severity of EAE in IRF7-deficient and control mice

	Incidence	Onset (day)	# mice with EAE that did not reach Grade 3	# mice reaching Grade 5^a^	# mice showing remission^b^
Wild-type	29/39	12.99 ± 1.0	11/29	4/29	7/29

IRF7-KO	28/30	12.07 ± 0.7	6/28	12/28	2/28

^a^: Progression to Grade 5 was in all cases rapid and resulted in euthanasia before day 18.

^b^: Reduction in severity of EAE by one grade was defined as remission.

### Increased immune cell entry into IRF7-deficient CNS

**Figure 3:**
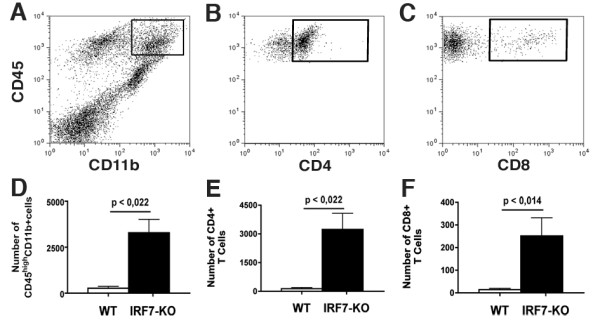
**IRF7 deficient mice had more CNS-infiltrating cells**. A-C) Example of FACS profiles of CNS from IRF7-KO mice with EAE. FACS profiles show CD45^high^CD11b^+ ^macrophages (A), CD4+ T cells (B), and CD8+ T cells (C). IRF7-KO mice (n = 7) had more infiltrating CD45^high^CD11b^+ ^macrophages (D), CD4+ (E) and CD8+ (F) cells in the CNS compared to WT mice (n = 6).

We then investigated the effect of IRF7 gene deletion on the infiltration of cells into the CNS. Flow cytometric analysis showed the clinical score of both WT and IRF7-deficient mice was correlated to the number of infiltrating blood-derived cells, as expected. Blood-derived CD45^high^CD11b^+ ^macrophages were discriminated by their higher level of expression of CD45 from CNS-resident microglia (Figure 3A). The total number of infiltrating CD45^high^CD11b^+ ^macrophages (p < 0.022, Figure 3A, D), CD4+ (p < 0.022, Figure 3B, E) and CD8+ T cells (p < 0.014, Figure 3C, F) was higher in IRF7-KO compared with WT CNS with more severe EAE.

### IRF7 deficiency affected distribution of infiltrating immune cells in CNS

**Figure 4:**
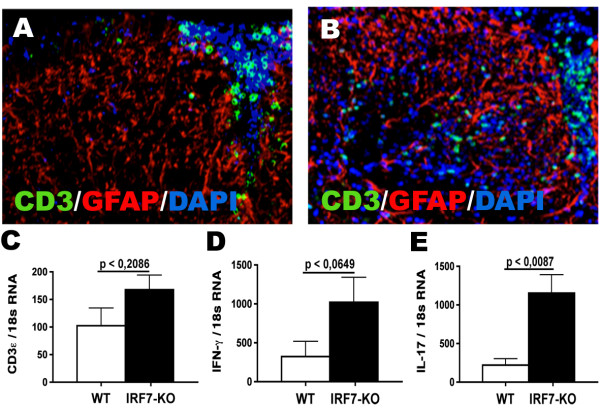
**CD3, GFAP, DAPI Immunostaining and quantitative real-time PCR analysis of T cells and related cytokines in IRF7-KO and WT mice with EAE**. CD3+ T cells were dispersed more diffusely in the spinal cord of IRF7-deficient mice with EAE (B) compared to the more focal infiltration pattern in WT spinal cord (A). IRF7 deficiency had no apparent effect on GFAP+ cells (astrocytes). A-B) original magnification 20×. C-E). CD3ε (C), IFN-γ (D), and IL-17 (E) gene expression in the CNS of IRF7-deficient (n = 6-7) and WT mice (n = 5-7) were calculated and normalized to 18s rRNA.

Fluorescence microscopy was used to localize infiltrating cells in the CNS. CD3+ T cells were increased in number and more diffusely dispersed in white matter in the spinal cord of IRF7-deficient mice with EAE, compared to the more focal and constrained infiltration pattern in WT spinal cord (Figure 4). In contrast to T cells, there was no apparent effect of IRF7-deficiency on numbers or distribution of GFAP+ astrocytes (Figure 4A, B). We further examined CD3ε, IFNγ and IL17 gene expression by quantitative RT-PCR. The content of CD3ε and IFNγ mRNA was increased in spinal cords from both IRF7-deficient and WT mice with EAE, but no significant differences could be measured when CD3ε (p < 0.2086) and IFN-γ mRNA (p < 0.0649) were compared between IRF-7 deficient and WT mice (Figure 4C, D). In contrast, IL-17 gene expression was higher (p < 0.0087) in IRF7-KO spinal cord than in spinal cords from WT mice with EAE (Figure 4E).

### Increased percentage of CD4+IFNγ+ T cells in LN of IRF7-deficient mice

**Figure 5:**
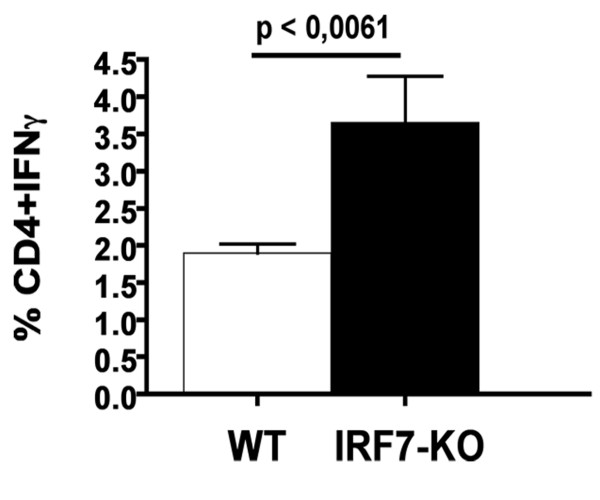
**IRF7 deficiency resulted in increased percentage of CD4+IFNγ+ T cells in LN**. After immunization with MOGp35-55 in CFA, IRF7-KO mice showed a significantly greater percentage of CD4+IFNγ cells in LN compared to similarly-immunized WT mice.

To further investigate the role of IRF7 on Th1 and Th17 cells during EAE, we measured IFNγ and IL17 production by CD4+ T cells from spinal cords and LN. IRF7 deficiency did not affect percentages of T cells producing these cytokines in spinal cord (not shown). However, lack of IRF7 resulted in an increase (p < 0.006) in the percentage of CD4+IFNγ+ cells (Figure 5), but not in CD4+IL17+ T cells (not shown) in LN.

### Elevated CCL2, CXCL10 and IL-1β expression in IRF7-deficient EAE

**Figure 6:**
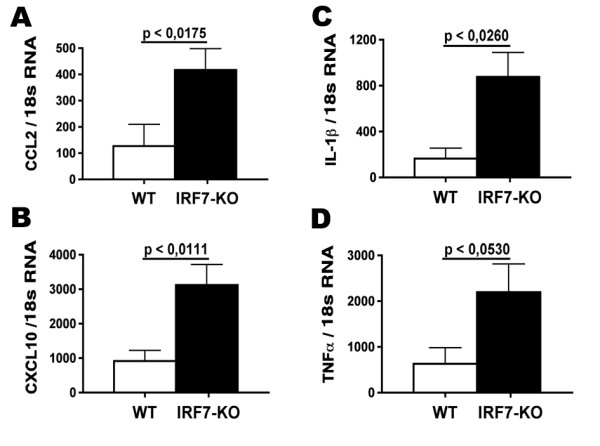
**Changes in cytokine and chemokine gene expression in IRF7 deficient (n = 6-7) and WT mice (n = 6-7)**. Real-time PCR analysis of CCL2 (A), CXCL10 (B), IL-1β (C) and TNF-α (D) showing these gene expression levels.

We next examined whether lack of IRF7 affected the expression of inflammatory mediators that are known to be involved in induction and regulation of EAE. Levels of the chemokines CCL2, CXCL10, and cytokines IL-1β and TNF-α increased in EAE and correlated to clinical severity (not shown). Quantitative RT-PCR analysis showed that the increases in expression of CCL2 (p < 0.0176, Figure 6A) and CXCL10 (p < 0.0111, Figure 6B), IL-1β (p < 0.0260, Figure 6C) and TNF-α (p < 0.0530, Figure 6D) were higher in IRF7-deficient spinal cord than in spinal cord from WT mice with EAE.

## Discussion

The pathogenesis of MS and its animal models include immune cell activation and their infiltration to the CNS, causing demyelination and axonal damage. The activation of immune cells involves innate receptor signaling [19], that is up-regulated in mice with EAE and modulates pathogenesis of EAE [20,21]. The innate receptor signaling that induces type I IFN, involves IRF7 [22]. IRF7 is activated by innate receptor signaling and regulates the induction of type I IFN. Type I IFN signaling further induces IRF7. It has been shown that the level of IFN-β in the CNS was increased in mice with EAE [13]. In concordance with this, we show that levels of IRF7 are increased with EAE severity. Thus, the increased IRF7 expression may represent a protective function for IRF7 in EAE.

The mechanism by which type I IFNs regulate CNS inflammation has been shown to include reduction of leukocyte migration to the CNS and inhibition of T cell responses [23,24]. Our findings support the former role.

IFN-β has been widely used in treatment of MS, and effects of type I IFN in animal models are generally similar to clinical findings. Oral administration of IFN-α to rats caused reduction of inflammation and ameliorated EAE symptoms [23]. Similarly, treatment with IFN-β could ameliorate EAE in rats and was associated with a reduction in the number of infiltrating leukocytes to the CNS [25,26]. In addition, IFN-β deficient mice develop more severe EAE with increased leukocyte infiltration [14]. This was supported by studies in IFNAR deficient mice that likewise showed more severe EAE with increased infiltration [13,20]. Here we show that IRF7 deficiency resulted in more severe EAE with higher leukocyte infiltration than WT mice with EAE. Our findings point to IRF7 as a key signaling intermediate in modulation of autoimmune demyelinating disease, and open the possibility that innate signals may also be protective.

Leukocyte infiltration is regulated by chemokines and cytokines and previous studies have shown that CCL2 and CXCL10 play a key role in attracting leukocytes to the CNS in both EAE and after brain injury [13,27,28]. In the present study we show that the increase in CCL2 and CXCL10 during EAE was significantly higher in IRF7 deficient mice compared with WT mice. The increased chemokine expression in the CNS might either be secondary to increased entry of peripheral immune cells, which are a potential source of several chemokines and cytokines, or could be due to direct effect of IRF7 on chemokine expression, or to a combination of the two. It has been demonstrated that innate receptor and type I IFN signaling regulate chemokine expression [22,29-31], but whether lack of IRF7 directly leads to increased chemokine and cytokine expression is not known. It has been shown that endogenous IFN-β selectively inhibits TNF-α, but not IL-1β expression [14], whereas in another study it was shown that IFN-β enhanced production of both [32]. It is also shown that deficiency in IFNAR resulted in reduced CXCL10 expression, but had no effect on CCL2 [8,31]. The effect of type I IFN signaling on production of chemokine and cytokines seems to depend on context.

However, in line with our observation in this study, Prinz et al., showed that CXCL10 and CCL2 levels in IFNAR-deficient mice increased [13]. CCL2 and CXCL10 chemokines are known to control both degree and pattern of leukocyte entry [33-35], and correspondingly we show that the increased chemokine level was associated with increased and more disseminated infiltration.

Taken together this could suggest that IRF7 regulates leukocyte infiltration through type I IFN regulated chemokine release. Alternatively, regulation could be mediated through IFN-independent mechanisms, by which IRF7 independently inhibits leukocyte migration, for instance by blocking the release of chemokines. IRF7 could also influence leukocyte entry to the CNS through mechanisms involving the inhibition of matrix metalloproteinase or adhesion molecules [36-38].

MS is considered to be a T cell mediated autoimmune disease. It has been shown that IFN-γ secreting Th1 and IL-17 secreting Th17 cells play a crucial role in EAE [39,40], although neither cytokine is absolutely required for EAE [41,42]. The ratio of Th17 and Th1 has been reported to be crucial for the localization of infiltration in CNS of mice with EAE [43]. It has been shown that innate receptor induced type I IFN is essential in limiting Th17 development and autoimmune inflammation [20,44]. In concordance, we find that IRF7 deficient mice had significantly higher number of T cells in the CNS compared with WT mice with EAE. Additionally, both IL17 and IFNγ were expressed at higher levels in the CNS of IRF7 deficient mice compared with WT. Our findings point to IRF7 as a key signaling intermediate in type I IFN modulation of autoimmune disease.

We used CXCR3 and CCR6 as surrogate markers for Th1 and Th17 respectively to show that IRF7 is expressed by both Th1 and Th17 CD4+ T cells and that IRF7 levels do not change significantly as mice progress through EAE. This suggests that both Th1 and Th17 could be affected by IRF7-deficiency. Consistent with this, both IFNγ and IL17 were increased in CNS. Galligan et al., and Guo et al., showed that IFNβ or IFNAR signaling inhibited Th17 development, and Guo et al., also showed that TRIF signaling inhibited both Th1 and Th17, consistent with our findings [15,20]. It also cannot be excluded that enhanced Th1 and Th17 responses in our study reflected altered activity of antigen-presenting cells, since macrophages, dendritic cells and microglia can all express CD11b and so were potentially affected by IRF7-KO. Dissection of the relevant cell type(s) whose IRF7 response controlled inflammatory T cell induction would require lineage-specific knockouts, such as were used by Prinz and colleagues [13]. We then performed intracellular cytokine staining of CD4+ T cells from LN and spinal cords. The percentages of CD4+IFNγ+ and CD4+IL17+ T cells in the spinal cords were unaffected by IRF7 deficiency. However, the percentages of CD4+IFNγ+ cells in the lymph nodes increased in IRF-KO mice. Our data had already shown increased expression of both IFNγ and IL17 in CNS of IRF7 KO mice, though only to significance for IL17. Modulation of Th1 development and IFNγ production by Type I IFN has been described by others [14,45,46]. The differences in data obtained from intracellular cytokine staining and cytokine message measured in spinal cords could be attributed to the fact that IL17 message detected by PCR in IRF7-KO could originate from sources other than T cells in the CNS.

EAE also involves gamma-delta T cells which do not express either CD4 or CD8 [47], and we can speculate that the lack of correspondence that we have shown between CD3 mRNA and CD4 and CD8 numbers may indicate differential effect of Type I IFN signaling on this subset. Alternatively it is possible that CD3 mRNA levels were downregulated in activated IRF7-KO T cells.

Genetic factors contribute to an individual's risk of developing autoimmune disease. The transcription factors IRF5 and IRF8 that are involved in both innate receptor and type I IFN signaling pathways have been identified as risk genes associated with MS [48,49]. IRF7 itself has been identified as a risk factor for human systemic lupus erythematosus [50] and has been shown to be co-regulated along with IRF8 in MS [49]. In the present study, we demonstrate functional significance of IRF7 in regulation of EAE. This would argue for loss of function as the probable basis for IRF7 as a risk factor in MS, although such association or its mechanism has not been established.

## Conclusion

Administration of IFN-β as therapy is beneficial for MS and it needs to be considered whether and how endogenous IFN I signaling would also contribute to disease modulation. Our results point to IRF7 as controlling the immunoregulatory effects of IFN-β and potentially acting to direct both innate and IFNAR signals towards regulatory pathways. This opens possibilities for a precise targeting of signaling pathways in MS. Future studies on treatment of MS may therefore consider IRF7 as therapeutic target.

## List of abbreviations

APC: allophycocyanin; CNS: central nervous system; EAE: experimental autoimmune encephalomyelitis; IRF7: Interferon regulatory factor 7; IFN: Interferon; IL-1β: interleukin-1β; IFNAR: type I interferon receptor; LN: lymph node; MOG: myelin oligodendrocyte glycoprotein; MS: multiple sclerosis; PE: phycoerythrin.

## Competing interests

The authors declare that they have no competing interests.

## Authors' contributions

TO and RK conceived and designed the experiments. MS, ML, JTM and RK performed the experiments. MS, ML, JTM, RK and TO analyzed the data. MS, ML, JTM, RK and TO wrote the paper. All authors have read and approved the final manuscript.

## Supplementary Material

Additional file 1 **Expression of IRF7 by cell subsets in LN**. IRF7 gene expression was measured by qRT-PCR in CXCR3+CD4+, CCR6+CD4+ and CD45+CD4-CD8- cells, sorted from LN of immunized mice. Days after immunization are shown on the × axis. No statistically significant differences in IRF7 gene expression were detected between these populations. Columns show means, error bars show SEM. ND: not detected. Click here for file
