# Supplementary figures and images for: Interferon regulatory factor-7 modulates experimental autoimmune encephalomyelitis in mice
Source: J Neuroinflammation. 2011 Dec 23;8:181. doi: 10.1186/1742-2094-8-181 (PMC3260126; doi:10.1186/1742-2094-8-181)

IRF7 / 18sRNA

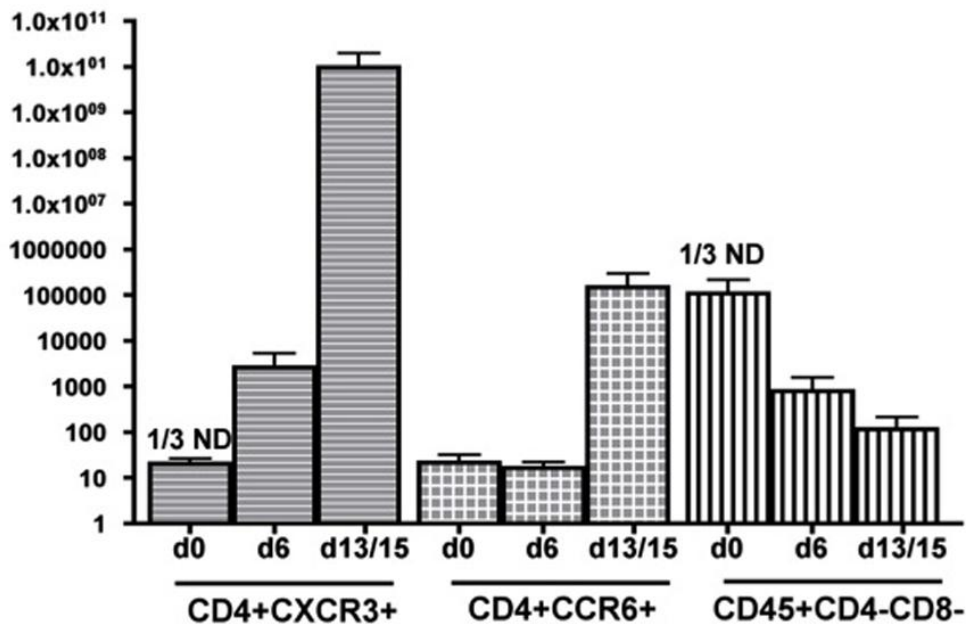

Supplement: Additional file 1 — Expression of IRF7 by cell subsets in LN. IRF7 gene expression was measured by qRT-PCR in CXCR3+CD4+, CCR6+CD4+ and CD45+CD4-CD8- cells, sorted from LN of immunized mice. Days after immunization are shown on the × axis. No statistically significant differences in IRF7 gene expression were detected between these populations. Columns show means, error bars show SEM. ND: not detected. [file 1742-2094-8-181-S1.PDF]
